# A late bloom at the apex: development of apical hypertrophic cardiomyopathy phenotype in advanced age

**DOI:** 10.1093/ehjcr/ytag723

**Published:** 2026-09-23

**Authors:** Sachin Sapkota, Muhammad Kasim Malik, Sandesh Murali, Gregory Milligan

**Affiliations:** Internal Medicine, Texas Tech University Health Sciences Center, 2000 B Woodrow Bean Transmountain Dr, Suite 200, El Paso, TX 79911, USA; Department of Cardiovascular Medicine, Baylor Scott & White—The Heart Hospital, 1100 Allied Dr, Plano, TX 75093, USA; Internal Medicine, Texas Tech University Health Sciences Center, 2000 B Woodrow Bean Transmountain Dr, Suite 200, El Paso, TX 79911, USA; Department of Cardiovascular Medicine, Baylor Scott & White—The Heart Hospital, 1100 Allied Dr, Plano, TX 75093, USA; Advanced Heart Failure & Transplant Cardiology, Baylor Scott & White—The Heart Hospital, 1100 Allied Dr, Plano, TX 75093, USA

**Keywords:** Hypertrophic Cardiomyopathy, CMR, Apical

## Summary

Hypertrophic Cardiomyopathy (HCM) is classically a disease of young and middle-aged, leading to assumptions that phenotypic development is unlikely in advanced age. We present cardiac MRI images demonstrating the definitive development of apical variant HCM in a patient's ninth decade of life. This case highlights the utility of serial multimodal imaging in identifying interval phenotypic changes.

## Case description

An 89-year-old woman with permanent atrial fibrillation, hypertension, heart failure with preserved ejection fraction (HFpEF), and prior transcatheter tricuspid valve repair presented with progressive exertional dyspnoea. Electrocardiographic and echocardiographic findings were concerning for interval development of apical left ventricular hypertrophy (see Supplementary material online, *Figure S1* and *Video S1*). Cardiac magnetic resonance (CMR) imaging revealed an apical hypertrophic cardiomyopathy (HCM) phenotype with marked apical hypertrophy (18.5 mm), elevated native T1, and extracellular volume, without the presence of late gadolinium enhancement (LGE). Cardiac magnetic resonance imaging 7 years prior demonstrated normal biventricular function and wall thickness, preserved ejection fraction, and no LGE (*Figure 1D–F*). Formal evaluation for an infiltrative aetiology was not performed given the absence of myocardial hyperenhancement on CMR.

**Figure 1:**
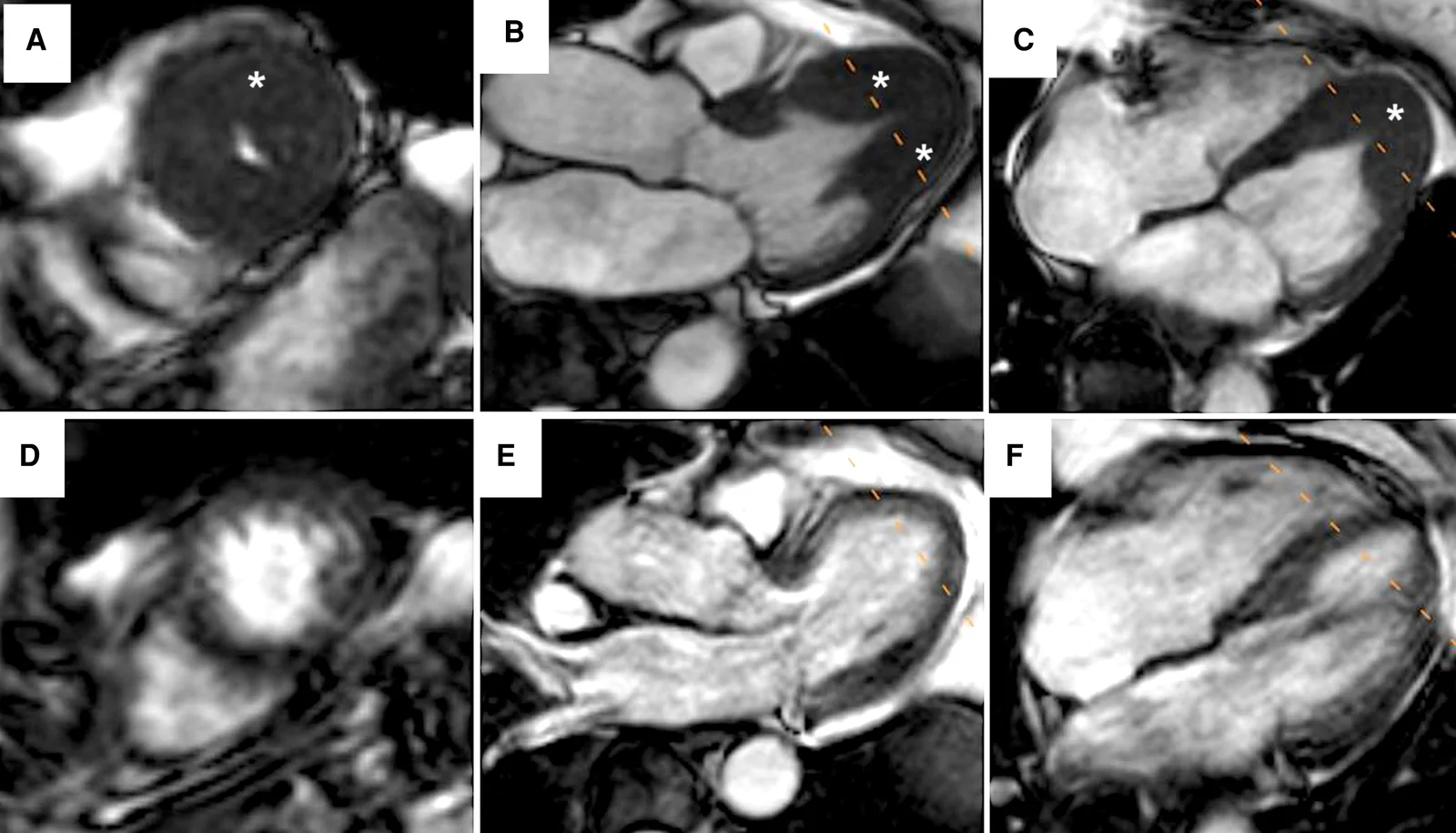
Interval development of severe apical left ventricular hypertrophy (white asterisks) on repeat cardiac magnetic resonance (CMR) imaging. (*A–C*) (age 89 years): steady-state free precession (SSFP) cine CMR images in apical short-axis (A), three-chamber (*B*), and four-chamber (*C*) views obtained at end-diastole. Dashed lines in long-axis windows denote the plane of short-axis acquisition. Maximum apical wall thickness, 18.5 mm (inferoseptal segment). Segmental end-diastolic wall thicknesses: anteroseptal basal/mid/distal, 12.9/14.2/18.1 mm; inferoseptum, 7.6/9.2/18.5 mm; anterior, 7.2/7.5/17.5 mm; inferior, 7.7/7.3/15.8 mm; lateral, 5.6/6.7/15.2 mm; inferolateral, 7.0/6.2/15.9 mm. Left ventricle (LV) volumes: left ventricular ejection fraction (LVEF), 57.9%; LV, mass 71.7 g (43.7 g/m^2^). Native T1, 1125 ms (reference 950–1050 ms); extracellular volume, 40% (reference ∼25–27%); T2, 57 ms (reference 45–58 ms); T2*, 33.9 ms (normal > 20 ms). No late gadolinium enhancement. No apical aneurysm, intracardiac thrombus, and midventricular obstruction. (*D–F*) (age 82 years): corresponding cine CMR images in similar planes. Biventricular function within normal limits, with mildly increased basal wall thickness (anteroseptum, 10.2 mm; posterolateral wall, 9.7 mm); LV end-diastolic dimension, 4.1 cm; and LVEF, 51.2%. No late gadolinium enhancement was identified. Parametric mapping was not performed on this study.

Adult-onset HCM has been previously described^1^; however, late onset within the ninth decade of life is exceedingly rare. Genetic testing identified a variant of uncertain significance in FHL1 (p.Lys30Arg). The FHL1 gene is not diagnostic of HCM but has been associated with X-linked HCM.^2^ Female carriers may exhibit variable expressivity through skewed X-inactivation, which may account for late phenotypic expression.

The 2024 American Heart Association/American College of Cardiology Guideline recommends for the serial reassessment of genetic variants to determine potential reclassification, which may impact on diagnostic accuracy and cascade genetic testing among family members.^3^ Although no age cut-off for familial phenotype screening is defined, the guideline notes that many clinicians continue surveillance until mid-life.^3^ The present case challenges this practice by demonstrating clinically significant manifestation at a more advanced age, highlighting the continued need for an individualized approach to patient care.

## Supplementary Material

ytag723_Supplementary_Data

## Data Availability

Relevant data are included within the article, and additional de-identified clinical information may be available from the corresponding author upon reasonable request, subject to institutional policies and approval.

## Supplementary material

**Consent:** The authors confirm that written consent for submission and publication of this case report, including images and associated text, has been obtained from the patient in line with the COPE guidelines.
